# Analysis of pectin mutants and natural accessions of *Arabidopsis* highlights the impact of de-methyl-esterified homogalacturonan on tissue saccharification

**DOI:** 10.1186/1754-6834-6-163

**Published:** 2013-11-18

**Authors:** Fedra Francocci, Elisa Bastianelli, Vincenzo Lionetti, Simone Ferrari, Giulia De Lorenzo, Daniela Bellincampi, Felice Cervone

**Affiliations:** 1 Dipartimento di Biologia e Biotecnologie “Charles Darwin”, Sapienza Università di Roma, Piazzale Aldo Moro 5, Rome 00185, Italy

**Keywords:** Saccharification, Plant cell wall, Pectin, Homogalacturonan, *Arabidopsis thaliana*

## Abstract

**Background:**

Plant biomass is a potentially important renewable source of energy and industrial products. The natural recalcitrance of the cell walls to enzymatic degradation (saccharification), which plants have evolved to defend themselves from biotic stresses, represents a major bottleneck for the industrial bioconversion of lignocellulosic biomasses. The identification of factors that influence the cell wall recalcitrance to saccharification may help to overcome the existing limitations that hamper the utilization of biomass.

**Results:**

Here we have investigated in *Arabidopsis thaliana* the impact of homogalacturonan (HG) content and structure on tissue saccharification. We characterized mutants affected in genes encoding proteins involved in HG biosynthesis (*quasimodo2-1*; *qua2-1*) and methylesterification (*pectin methylesterase 3*; *pme3*). We also analyzed the natural variation of *Arabidopsis* through the characterization of a nested core collection of 24 accessions generated to maximize genetic variability. We found a negative correlation between the level of de-methyl-esterified HG (HGA) and cellulose degradability.

**Conclusions:**

We propose to use the level of HGA domains as a biochemical marker of the cell wall recalcitrance to saccharification. This may be utilized for selecting, on a large scale, natural variants or mutants with improved bioconversion features.

## Background

Liquid fuels derived from plant biomass are a promising, renewable alternative to fossil fuels. Presently, most bioethanol is produced by fermentation of sucrose or starch deriving from food crops such as sugarcane, corn, and wheat (first generation bioethanol) [1]. However, competition between the increasing needs for food and fuel, discontinuous feedstock availability, and high production costs pose serious socioeconomic and logistical problems that hamper the large-scale adoption of this energy source. A suitable alternative to the use of food crops is the abundant and low cost lignocellulosic biomass derived from dedicated energy crops or from agricultural wastes (second generation bioethanol) [2]. A key step in the bioconversion of lignocellulosic biomass is saccharification, that is, the degradation of the plant cell wall polysaccharides into soluble sugars that can be used by microorganisms for fermentation. Currently, enzymatic hydrolysis is the most promising and environmentally friendly technology available for saccharification [3]. However, a major bottleneck for the industrial implementation of biomass utilization is the natural recalcitrance of the plant cell walls to enzymatic hydrolysis [4]. The use of plants with walls less recalcitrant to hydrolysis may not only improve the saccharification process but also reduce the need of costly pre-treatments. The heterogeneity and complexity of the cell wall and the inter-polymeric interactions of its structural components are major factors contributing to recalcitrance [5]. For example, cellulose microfibrils are tethered by xyloglucan through hydrogen bonds, while hemicelluloses interact with pectin via both non-covalent and covalent bonds [6]. Crystallinity of cellulose as well as branches of xylan also influence enzymatic saccharification [7,8], and lignification strongly reduces cellulose accessibility to degrading enzymes [9]. The reduction of the level of some lignin precursors in transgenic alfalfa plants significantly increases the efficiency of tissue saccharification. Besides lignin, other cell wall components may be considered as suitable targets for the development of novel varieties more amenable to saccharification [5]. Pectin is one of these components because it influences permeability and adhesive properties of the cell wall, and its position in the wall is crucial for the accessibility of cellulose and other polysaccharides to cell wall-degrading enzymes (CWDEs) [4,10]. The backbone of pectin is homogalacturonan (HG), which consists of α-1,4-linked galacturonic acid (GalUA) residues partially methyl-esterified in C_6_[11]. HG is exported to the cell wall in a highly methylated form and undergoes a selective de-methylation by apoplastic pectin methylesterases (PMEs) [12]. The negatively charged carboxyl groups of adjacent HG chains, produced by PME activities, can form rigid calcium-mediated crosslinks that stiffen the cell wall [13]. De-methyl-esterified HG (henceforth HGA) may affect assembly of the cellulose network, influencing the deposition and alignment of microfibrils in the wall [14]. We have generated plants with a reduced content of HGA either by expressing a fungal polygalacturonase (PG plants) [15] or by overexpressing the PME inhibitors (PMEI plants) [16]. In both cases, transgenic plants exhibit significantly higher efficiency of enzymatic saccharification [17].

As an alternative to the genetic transformation approach, in the present work we evaluated whether mutations in *Arabidopsis* genes encoding proteins that influence the level of HGA affect the cell wall recalcitrance to cellulase hydrolysis. In particular, we analyzed the mutant *quasimodo2-1* (*qua2-1*) that carries a mutation in a putative pectin methyltransferase (PMT) and shows defects in cell adhesion and growth [18], and a knock-out mutant *pectin methylesterase 3* (*pme3*) of a PME isoform that affects the level of pectin methylesterification in leaves [19]. In addition, we characterized, in terms of content of HGA and saccharification efficiency, a polymorphic nested core collection of *Arabidopsis* accessions [20]. Our study shows a negative correlation between the level of HGA regions and cellulose degradability, indicating that HGA level is a trait that may be exploited for a wide-scale selection and breeding of plants for biofuel production.

## Results and discussion

### Tissues of mutants with a reduced HGA level have higher cellulose degradability

**Figure 1:**
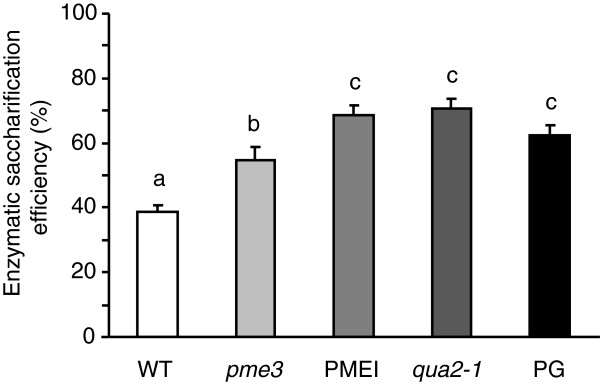
**Saccharification efficiency of WT, *pme3*, PMEI, *qua2-1, *and PG leaves.** Saccharification efficiency was measured after 24 hours of treatment with 0.5% Celluclast. Bars represent average of saccharification efficiency ± SE (n ≥4). Different letters indicate statistically significant differences, according to ANOVA followed by Tukey’s test (*P* <0.05). The experiments were repeated at least three times with similar results. PG, polygalacturonase; *pme3*, *pectin methylesterase 3*; PMEI, pectin methylesterase inhibitor; *qua2-1*, *quasimodo2-1*; SE, standard error; WT, wild type.

*Arabidopsis* mutants, *qua2-1* and *pme3*, respectively affected in the HG content and methylesterification (Additional file 1: Figure S1) [18,19], were compared to transgenic PG plants and PMEI plants previously demonstrated to have, respectively, a lower HG content and a higher degree of methylesterification (DM) [17]. Leaf tissues were treated with Celluclast 1.5 L (Sigma-Aldrich, St Louis, MO, USA), a commercial preparation that contains mostly cellulose-degrading activities [21]. Saccharification efficiency was measured as the percentage of sugars released after enzymatic hydrolysis with respect to total sugars quantified in the tissues. Significant differences were observed among the different genotypes (Figure 1). A 43% higher saccharification efficiency with respect to the wild type (WT) was observed by using leaves of *pme3* plants, while saccharification of leaves of the *qua2-1* mutant and of PG and PMEI plants was about 75% higher than that of WT plants. The monosaccharide compositions of the enzymatic hydrolysates of leaves from mutants and transgenic plants mainly revealed a higher content of glucose as compared to the glucose released from leaves of WT plants (Table 1). This indicated that cellulose in the cell wall of mutants and transgenic plants is more accessible to cellulase degradation.

**Table 1:** Analysis of monosaccharides released after enzymatic hydrolysis of leaves from transgenic and mutant plants

	**Ara**^**a**^	**Gal**^**a**^	**Glc**^**a**^	**Xyl**^**a**^
WT	0.11 ± 0.05	0.24 ± 0.04	0.99 ± 0.57	0.04 ± 0.02
*pme3*	0.11 ± 0.00	0.28 ± 0.03	2.18 ± 0.32*	0.05 ± 0.01
*qua2-1*	0.14 ± 0.01	0.21 ± 0.02	1.56. ± 0.05*	0.03 ± 0.0
PMEI	0.14 ± 0.01	0.30 ± 0.06	2.22 ± 0.07*	0.04 ± 0.01
PG	0.13 ± 0.00	0.21 ± 0.01	1.56 ± 0.09*	0.05 ± 0.01

^a^nmoles per μg of total sugar released after 24 hours of hydrolysis from WT (Col-0), *pme3, qua2-1*, PMEI*,* and PG plants, quantified using HPAEC-PAD. Values are expressed as nmoles of each sugar per μg of total sugars (TS) in the tissues. Results are means ± SD of three biological replicates. Asterisk (*) indicates statistically significant differences with respect to the WT according to Student’s *t*-test (*P* <0.05). The experiments were repeated at least three times with similar results. Ara, arabinose; Col-0, Columbia-0; Gal, galactose; Glc, glucose; HPAEC-PAD, high-performance anion-exchange chromatography with pulsed amperometric detection; PG, polygalacturonase; *pme3*, *pectin methylesterase 3*; PMEI, pectin methylesterase inhibitor; *qua2-1*, *quasimodo2-1*; WT, wild type; Xyl, xylose.

**Figure 2:**
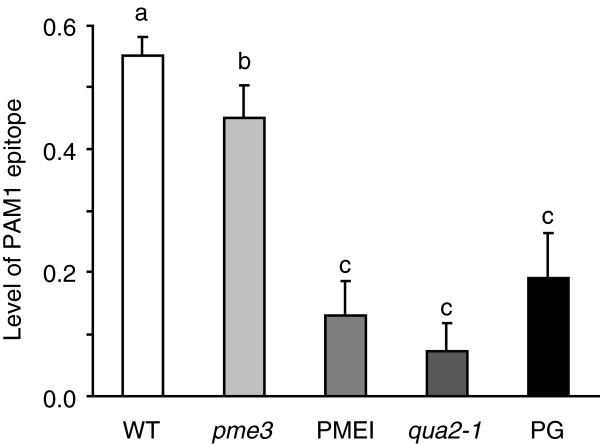
**Variability of content of HGA regions in *Arabidopsis *mutants affected in pectin composition.** The levels of HGA domains were determined by immunodot assay on ChASS fraction extracted from leaves of 4-week-old *Arabidopsis* WT (Col-0), mutants (*qua2-1* and *pme3*), and transgenic plants (PG and PMEI). Equal amounts of ChASS were hybridized with the monoclonal antibody PAM1. PAM1 epitope levels were normalized with respect to the standard polygalacturonic acid (PGA). Bars represent means of PAM1 level ± SD (n = 3). Different letters indicate statistically significant differences, according to ANOVA followed by Tukey’s test (*P* <0.05). Similar results were obtained in at least three other experiments. ChASS, chelating agent-soluble solids; Col-0, Columbia-0; HGA, de-methyl-esterified homogalacturonan; PG, polygalacturonase; PGA, polygalacturonic acid; *pme3, pectin methylesterase 3*; PMEI, pectin methylesterase inhibitor; *qua2-1*, *quasimodo2-1*; WT, wild type.

**Figure 3:**
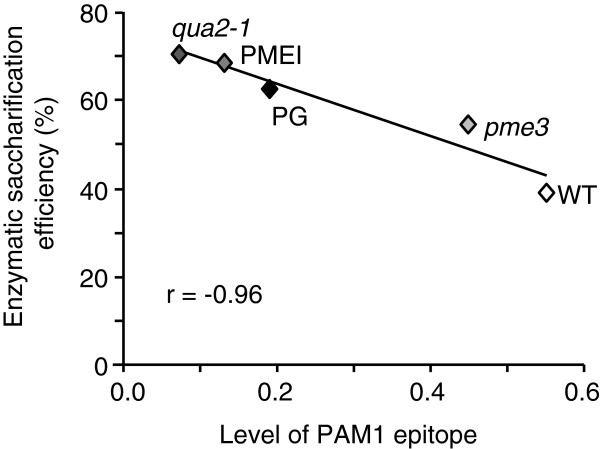
**Scatter plot between PAM1 epitope level and enzymatic saccharification efficiency.** Correlation between PAM1 level and saccharification efficiency was examined based on the Pearson correlation efficiency. A strong negative correlation was found (*r* = -0.96) among the genotypes analyzed. PG, polygalacturonase; *pme3*, *pectin methylesterase 3*; PMEI, pectin methylesterase inhibitor; *qua2-1*, *quasimodo2-1*; WT, wild type.

Biomass yield is one important target for improving programs dedicated to biofuels [22]. The biomass of *qua2-1* plants was significantly lower than that of WT plants (Additional file 2: Table S1), as was the biomass of PG plants [17]. Both kinds of plants have a lower content of HG and this may negatively affect biomass production. *Pme3* mutants, instead, did not differ from the WT plants in terms of biomass production and exhibited an improved saccharification efficiency, likely due to a reduced content of HGA like in the case of PMEI plants [17,19]. The level of HGA domains was measured in the chelating agent-soluble solid (ChASS) fractions of both mutants and transgenic plants by immunodot assay using the monoclonal antibody PAM1, which specifically recognizes large blocks of HGA (at least 30 contiguous GalUA units) [23,24]. The lowest level of PAM1-binding epitopes (about 80% less than in WT plants) was observed in the ChASS fraction of the *qua2-1* mutant (Figure 2). A significantly lower level of PAM1 epitopes was also detected in the ChASS fraction from PG plants and PMEI plants (about 65% and 76%, respectively) as well as in *pme3* plants (about 20% with respect to the WT). These results indicate that a reduced level of PAM1 epitopes may result either from the reduction of the overall HG content as in the case of *qua2* and PG plants or from an increase of the degree of pectin methylesterification as in the case of *pme3* and PMEI plants. In both cases, the improvement of saccharification efficiency is achieved, likely due to an increased accessibility of cellulose to cellulases. Pearson correlation analysis between the abundance of PAM1-reactive epitopes in the ChASS fraction of the tested genotypes and the saccharification efficiency of their leaf tissues resulted in a significantly negative value (*r* = -0.96) (Figure 3). We concluded that, in addition to the transgenic approach, natural or targeted mutations of key genes regulating the biosynthesis or the *in muro* modification of HG can be utilized to improve tissue saccharification. Possible targets are PMTs and PMEs, but also pectin galacturonosyltransferases (GAUT), GAUT-like (GATL), and PMEIs may be considered.

### *Arabidopsis* natural accessions with a low level of HGA show reduced recalcitrance to enzymatic hydrolysis

**Figure 4:**
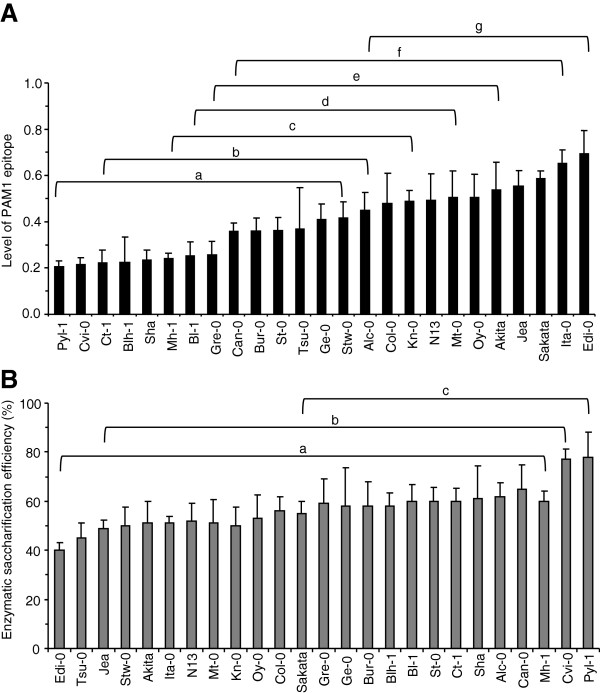
**Analysis of the levels of HGA regions and saccharification efficiency in the *Arabidopsis *nested core collection. (A)** PAM1 epitope content. Bars represent average of PAM1 epitope level ± SD (n = 3) express with respect to 1 μg PGA signal. **(B)** Saccharification efficiency of leaves. Bars represent average of saccharification efficiency ± SD (n ≥10). Different letters indicate statistically significant differences, according to ANOVA followed by Tukey’s test (*P* <0.05). The experiments were repeated three times with similar results. HGA, de-methyl-esterified homogalacturonan; PGA, polygalacturonic acid.

**Figure 5:**
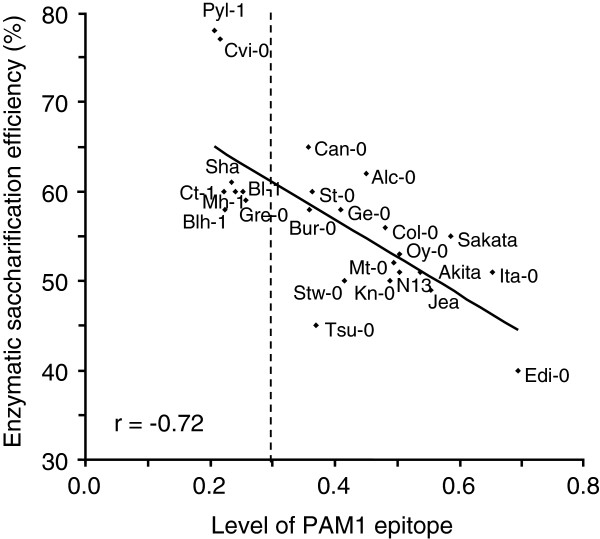
**Scatter plot of correlation analysis between enzymatic saccharification efficiency and PAM1 level in *Arabidopsis *natural accessions.** PAM1 epitope levels were determined by immunodot assay (express with respect to 1 μg PGA signal). Enzymatic saccharification efficiency was measured after 24 hours of treatment with 0.5% Celluclast. Pearson correlation analysis revealed a high negative correlation (*r* = -0.72) between saccharification efficiency and PAM1 content among the accessions analyzed. PGA, polygalacturonic acid.

The relationship between the level of HGA and susceptibility to enzymatic saccharification was also explored in a nested core collection of 24 *Arabidopsis* accessions (Additional file 3: Table S2), rationally generated to maximize, with minimum repetitiveness, the genetic diversity present in a large collection of 265 natural accessions [20]. The Columbia-0 (Col-0) genotype, which is the genetic background of all mutant and transgenic plants used in this work, was added to the nested core collection. A detailed characterization of pectin polysaccharides was performed by determining uronic acid content and DM in leaf cell walls of the different accessions. The analyses showed a low variability in the uronic acid content (coefficient of variation (CV) = 0.12) and higher variability of the cell wall DM (CV = 0.28) (Additional file 4: Figure S2A, B). A large variability (CV = 0.38) was also observed in the biomass production of the accessions (Additional file 3: Table S2) as previously described [20]. Immunodot analysis of the ChASS fraction of leaf cell walls using PAM1 antibodies revealed a large variability in the content of PAM1-binding epitopes (CV = 0.36) with relative signals ranging from 0.2 to 0.7 and grouping in various classes of significance with different levels of PAM1 epitope (Figure 4A). The genotypes Pyl-1 and Edi-0 showed extreme values of 0.2 and 0.7, respectively, whereas Col-0 displayed a medium value of 0.48. Despite the different PAM1 epitope levels, Pyl-1 and Edi-0 exhibited a similar GalUA content (Additional file 5: Figure S3). The higher PAM1 epitope level exhibited by Edi-0 can be explained by the observed low DM in the cell wall of these plants (Additional file 4: Figure S2B). Enzymatic saccharification efficiency was determined in leaf tissues isolated from each accession. The results revealed significant differences but a lower variability among the genotypes (CV = 0.15) as compared to that of the PAM1 epitope level (Figure 4B). The extreme values were 40% (Edi-0) and 78% (Pyl-1), whereas Col-0 had intermediate saccharification efficiency (56%). The lower variability is possibly due to the multiple and compensatory action of different factors affecting the cell wall recalcitrance to enzymatic hydrolysis; that is, not only the structure of pectin, but also the structure and content of hemicellulose, cellulose, and lignin, and their interactions. However, Pearson correlation analysis between the levels of HGA regions and enzymatic saccharification efficiency resulted in an *r* value of -0.72, indicating a strong negative correlation between the two variables (Figure 5). Interestingly, the accessions with a level of PAM1 epitope lower than 0.3 exhibited the lowest cell wall recalcitrance to cellulase, with an efficiency of saccharification higher than the median value (57%) (Figure 5). This suggests that, below a threshold level of 0.3, the effects of PAM1 content on the cell wall digestibility by cellulase become prevalent and overcome those mediated by other cell wall components. Pyl-1 showed the greatest saccharification efficiency and the lowest PAM1 level, whereas Edi-0 showed the lowest saccharification efficiency and the highest PAM1 epitope level.

A correlation matrix performed using different pectin-related biochemical parameters, determined for the entire *Arabidopsis* collection, revealed a significant score between saccharification efficiency and PAM1 levels (Table 2), while the HG content and pectin DM are traits that cannot be considered for selection of varieties with good saccharification efficiency.

**Table 2:** **Correlation matrix between saccharification efficiency, biomass production, and pectin biochemical traits in *Arabidopsis *** **accessions**

	**PAM1**	**SE%**	**DM%**	**UA/AIS**	**ChASS/AIS**	**FW**	**DW/FW**
**PAM1**	1.00						
**SE%**	**-0.72**	1.00					
**DM%**	-0.31	0.55	1.00				
**UA/AIS**	0.02	-0.14	-0.22	1.00			
**ChASS/AIS**	0.33	-0.47	-0.36	0.00	1.00		
**FW**	0.26	-0.51	-0.36	0.35	0.51	1.00	
**DW/FW**	-0.06	0.07	0.06	-0.17	0.08	-0.31	1.00

Pearson correlation matrix determined from the 24 accessions and the ecotype Col-0 for enzymatic saccharification efficiency (SE%), biomass production (FW; DW/FW), PAM1 epitope level (PAM1), degree of methylesterification (DM%), uronic acid content/mg of AIS (UA/AIS), ratio between pectin fraction (μg sugars), mg of cell wall (ChASS/AIS), and biomass parameter (FW and DW). The only significant correlation is indicated in bold. AIS, alcohol-insoluble solids; ChASS, chelating agent-soluble solids; Col-0, Columbia-0; DM, degree of methylesterification; DW, dry weight; FW, fresh weight; SE, saccharification efficiency; UA, uronic acid.

## Conclusions

The results show that a low level of HGA in the cell wall correlates with a high enzymatic degradability of cellulose in *Arabidopsis*. A positive correlation between low level of HGA and high enzymatic saccharification has also been previously reported in tobacco and wheat plants [17]. This is consistent with the concept that HGA is the domain of pectin that, by forming crosslinks with calcium ions, acts as a ‘glue’ and keeps the wall polysaccharides together [25]. In order to deconstruct the plant cell wall, pathogenic microorganisms have a temporal strategy of producing CWDEs and secrete pectinases before other CWDEs [26]. The hydrolysis of pectin, therefore, is a prerequisite that makes the other cell wall components more accessible to CWDEs. We have shown that tissues of plants with a reduced content of acidic HGA domains are more efficiently digested by cellulase, likely because the accessibility of the enzyme to its substrate is facilitated in these plants. Our analysis of *qua2-1* and *pme3* mutants, and of *Arabidopsis* natural accessions, reinforces and validates the concept, and shows that among the pectin-related biochemical parameters the PAM1 epitope level correlates very well to the saccharification potential. The large variability of PAM1-binding epitopes exhibited by the nested core collection may have an adaptive significance. The existence of the variability in the model plant *Arabidopsis* suggests that the content of HGA may also be variable in other plant species. Therefore, the HGA level may be a suitable marker to identify crop plants with better characteristics of bioconversion. The use of a simple assay to measure the PAM1 epitope by immunodetection potentially makes this trait useful for high-throughput screenings.

## Material and methods

### Plant material and growth condition

*Arabidopsis* (*Arabidopsis thaliana*) Col-0 WT seeds were purchased from Lehle Seeds (Round Rock, TX, USA)*.* The production of transgenic PMEI (line 7) and PG (line 57) plants, and the selection of the *pme3* and *qua2-1* mutants, have been previously described [17-19]. All mutant lines used in the present work are in the Col-0 background. The 24-accession core collection was provided by the *A. thaliana* Genomic Resource Centre at National Institute for Agricultural Research (INRA), Versailles, France [20]. All the plants were grown in a growth chamber maintained at 22°C and 70% relative humidity under a 16-hour light/8-hour dark photoperiod (photosynthetic photon flux density; PPFD) of 100 μmoles photons m^-2^ s^-1^). Before harvesting, all plants were maintained for 24 hours in darkness to minimize starch accumulation. Fully expanded *Arabidopsis* rosette leaves were collected at 3.9 growth stage (http://www.arabidopsis.org/portals/education/growth.jsp).

### Enzymatic hydrolysis, sugar analysis, and determination of saccharification efficiency

Leaves were sterilized in a 1% sodium hypochlorite solution for 5 minutes and washed twice with sterile water. One hundred mg (wet weight) of leaf tissues from transgenic and ecotypes were cut into 0.25 cm^2^ square pieces and incubated at 37°C in a 5 mL filter-sterilized solution containing 50 mM sodium acetate buffer (pH 5.5), 0.02% NaN_3_, and 0.5% (vol/vol) Celluclast 1.5 L (cellulase from *Trichoderma reesei* ATCC 26921; 60.3 filter paper unit (FPU)/mL; product number C2730; Sigma-Aldrich, St Louis, MO, USA). The FPU for cellulase activity of Celluclast 1.5 L, quantified according to the standard method [27], was 60.3 FPU/mL. The enzymatic loading was 15 FPU/g leaf tissues. The leaf tissue was infiltrated under vacuum for 5 minutes. The incubation medium from leaves was collected after 24 hours of enzymatic treatment, centrifuged for 2 minutes at 11,000 × g, and the supernatant was then transferred into fresh tubes. The determination of carbohydrates in the enzymatic hydrolysates was performed according to the analytical procedure of the National Renewable Energy Laboratory (NREL) [27]. Enzymatic saccharification efficiency was determined as the percentage of sugars released by enzymatic hydrolysis from the amount of sugars present in the tissue prior to enzymatic hydrolysis. The amount of sugars present in the supernatant and total sugars present in the tissues was determined spectrophotometrically by phenol-sulfuric acid assay [28] using D-Glc to perform the standard calibration curve. Statistical analyses were performed using the ANOVA procedure of the software package STATISTICA (1999; StatSoft, Tulsa, OK, USA).

Monosaccharide composition of the enzymatic hydrolysates was determined by high-performance anion-exchange chromatography with pulsed amperometric detection (HPAEC-PAD; Ion Chromatography System ICS-3000, Dionex, Sunnyvale, CA, USA) with a 3 × 150 mm CarboPac PA20 column (Dionex). Before injection of each sample, the column was washed with 200 mM NaOH for 10 minutes and equilibrated with 6 mM NaOH for 10 minutes. Samples were subjected to an isocratic elution with 6 mM NaOH at a flow rate of 0.32 mL min^-1^ for 20 minutes. Monosaccharides were detected using a gold pulsed amperometric detector set on waveform A, according to the manufacturer’s instructions. Peaks were identified and quantified by comparison to a standard mixture of arabinose (Ara), galactose (Gal), glucose (Glc), and xylose (Xyl) (Sigma-Aldrich).

### Isolation of alcohol-insoluble solids (AIS) and degree of methylesterification (DM)

Extraction of AIS and DM analysis were performed as previously described [29] with minor modifications. Starch was removed by treating the AIS with the porcine Type I-A *α*-amylase (100 U g^-1^ AIS; product number A4268; Sigma-Aldrich) in a 100 mM potassium phosphate buffer pH 7., 5 mM NaCl, and 0.02% (w/v) NaN_3_ for 24 hours at 37°C. The suspension was centrifuged at 25,000 × *g* for 20 minutes, then washed with distilled water and 80% acetone.

### Extraction of chelating agent-soluble solids (ChASS) and immunodot assay

ChASS fractions were extracted from 10 mg of AIS for 4 hours at 70°C in a buffer containing 50 mM Tris-HCl, 50 mM ammonium oxalate, and 50 mM trans-1,2-cyclohexanediaminetetraacetic acid (CDTA) (pH 7.2). Pectin was precipitated by adding absolute ethanol to a final concentration of 30% to the supernatant and incubated at 4°C overnight. The samples were centrifuged at 25,000 × g for 20 minutes and suspended in water. The concentration of sugars in the ChASS fractions was spectrophotometrically determined with the phenol-sulfuric acid assay using D-GalUA as standard [28]. Extraction of ChASS was performed from two fully expanded leaves per plant. For each genotype three independent replicates were analyzed. For each experiment, ChASS fractions were applied as 1 μL aliquots to nitrocellulose membrane (0.45 μm pore size; Bio-Rad, Hercules, CA, USA) in a threefold dilution series (10, 3, and 1 μg/μL). Polygalacturonic acid (PGA; P-3889; lot number 68 F08231; Sigma-Aldrich) was used as standard and also applied as 1 μL aliquots in a dilution series (3, 1, and 0.3 μg/μL). Arrays were incubated for 1 hour in 5% (w/v) milk protein (MP; Bio-Rad) in PBS pH 7.8 (MP-PBS), and probed for 1.5 hours with primary PAM1ScFv (PAM1) monoclonal antibodies (purchased from PlantProbes, Paul Knox Cell Wall Lab, University of Leeds, Leeds, UK) [30] diluted 1:50 in 3% MP-PBS. After extensive washes in PBS, arrays were incubated with anti-His conjugated to horseradish peroxidase (A7058; Sigma-Aldrich) diluted 1:500 in MP-PBS buffer. After washing in PBS, arrays were developed using 4-chloro-1-naphthol as previously described [31]. A representative antibody array is shown in Additional file 6: Figure S4. Densitometric analyses of the dot signals were obtained using ImageJ software (http://rsbweb.nih.gov/ij/). The PAM1 signal per μg of samples was normalized with respect to PAM1 signal per μg of PGA. For each experiment, the PAM1 signals relative to the nine individual spots relative to the single genotype were averaged and expressed as the level of PAM1 epitope. Statistical analyses were performed using the ANOVA procedure using the software package STATISTICA.

### Monosaccharide composition of ChASS

ChASS was extracted as described above from AIS saponified with 80 μL of 0.25 M NaOH and neutralized with HCl after 1 hour of incubation at room temperature. ChASS was then hydrolyzed with 2 M trifluoroacetic acid (TFA) for 90 minutes at 120°C and dried under N_2_ gas stream. The monosaccharide composition of the pectin-enriched fraction was determined by HPAEC-PAD as described above. Peaks were identified and quantified by comparison to a standard mixture of rhamnose (Rha), Ara, fucose (Fuc), Gal, Glc, mannose (Man), Xyl, GalUA, and glucuronic acid (GlcUA) (Sigma-Aldrich).

## Abbreviations

ANOVA: Analysis of variance; AIS: Alcohol-insoluble solids; Ara: Arabinose; CDTA: Trans-1,2-cyclohexanediaminetetraacetic acid; ChASS: Chelating agent-soluble solids; Col-0: Columbia-0; CV: Coefficient of variation; CWDE: Cell wall-degrading enzyme; DM: Degree of methylesterification; DW: Dry weight; FPU: Filter paper unit; Fuc: Fucose; FW: Fresh weight; Gal: Galactose; GalUA: Galacturonic acid; GAUT: Galacturonosyltransferases; Glc: Glucose; GlcUA: Glucuronic acid; HG: Homogalacturonan; HGA: de-methyl-esterified homogalacturonan; HPAEC-PAD: High-performance anion-exchange chromatography with pulsed amperometric detection; INRA: National Institute for Agricultural Research; Man: Mannose; MP: Milk protein; NREL: National Renewable Energy Laboratory; PBS: Phosphate-buffered saline; PG: Polygalacturonase; PGA: Polygalacturonic acid; PME: Pectin methylesterase; pme3: *pectin methylesterase 3*; PMEI: Pectin methylesterase inhibitor; PMT: Pectin methyltransferase; PPFD: Photosynthetic photon flux density; qua2-1: *quasimodo2-1*; Rha: Rhamnose; TFA: Trifluoroacetic acid; WT: Wild type; Xyl: Xylose.

## Competing interests

The authors declare that they have no competing interests.

## Authors’ contributions

FF, EB, VL, DB, FC, and GDL designed the research. FF, EB, and VL performed the research. FF, EB, VL, DB, FC, GDL, and SF analyzed data. FF, EB, VL, DB, and FC wrote the manuscript, and GDL and SF edited the manuscript. All authors read and approved the final manuscript.

## Supplementary Material

Additional file 1: Figure S1 Monosaccharide composition of the ChASS fraction of cell walls from WT (Col-0), *qua2-1*, *pme3*, PG, and PMEI plants. Monosaccharide composition was determined by HPAEC-PAD. Values are expressed in mol% for each monosaccharide. Bars represent means ± SE (n = 4). Different letters indicate statistically significant differences in each monosaccharides between mutants or transgenic plants and WT plants, according to ANOVA followed by Tukey’s test (*P* <0.05). ChASS, chelating agent-soluble solids; Col-0, Columbia-0; HPAEC-PAD, high-performance anion-exchange chromatography with pulsed amperometric detection; PG, polygalacturonase; *pme3*, *pectin methylesterase 3*; PMEI, pectin methylesterase inhibitor; *qua2-1*, *quasimodo2-1*; SE, standard error; WT, wild type. Click here for file

Additional file 2: Table S1 Effect of *qua2-1* and *pme3* mutations on plant biomass. Fresh weight (FW) of aerial vegetative portion of plants and dry weight (DW/FW) ratio were calculated. Data represent the average ± SD of at least six plants. Asterisks indicate statistically significant differences between mutants and the WT according to Student’s *t*-test (*P* <0.05). DW, dry weight; FW, fresh weight; *pme3*, *pectin methylesterase 3*; *qua2-1*, *quasimodo2-1*; WT, wild type. Click here for file

Additional file 3: Table S2 Information about ecotypes included in the nested core collection from INRA including Col-0 and biomass yield. The Versailles identification number (AV), the name of accession, its geographical location, fresh weight (FW) of aerial vegetative portion of plants, and dry weight (DW)/FW ratio are reported. Data represent the average ± SD of at least six plants. Average, standard deviation, and coefficient of variation (CV) calculated from the entire collection are reported. Col-0, Columbia-0; CV, coefficient of variation; DW, dry weight; FW, fresh weight; INRA, National Institute for Agricultural Research. Click here for file

Additional file 4: Figure S2 Quantification of uronic acids and degree of methylesterification (DM) in cell walls of the *Arabidopsis* core collection. (A) Uronic acid content and (B) degree of methylesterification (DM) were analyzed in cell walls extracted from 4-week-old fully expanded leaves. Bars represent averages ± SD (n = 10). Different letters indicate statistically significant differences, according to ANOVA followed by Tukey’s test (*P* <0.05). DM, degree of methylesterification. Click here for file

Additional file 5: Figure S3 Monosaccharide composition of the ChASS fraction of cell walls from Col-0, Edi-0, and Pyl-1. Monosaccharide composition was determined by HPAEC-PAD. Values are expressed in mol% for each monosaccharide. Bars represent means ± SE (n = 4). Different letters indicate statistically significant differences in each monosaccharides of Edi-0 and Pyl-1, with respect to Col-0 according to ANOVA followed by Tukey’s test (*P* <0.05). ChASS, chelating agent-soluble solids; Col-0, Columbia-0; HPAEC-PAD, high-performance anion-exchange chromatography with pulsed amperometric detection; SE, standard error. Click here for file

Additional file 6: Figure S4 ChASS arrays using PAM1 antibodies. **(A)** Representative arrays relative to different *Arabidopsis* accessions are shown. The numbers represent three independent replicates for each accession. ChASS fractions were applied as 1 μL aliquots in a threefold dilution series (10, 3, and 1 μg/μL). As standard, PGA was also applied as 1 μL aliquots in a dilution series (3, 1, and 0.3 μg/μL). **(B)** Schematic representation of quantification steps of PAM1 signal, using the immunoblot for the N13 accession **(A)**. ImageJ software was used to quantify spot signals. The optical density of each spot was quantified as the peak area and defined as PAM1 signal. ChASS, chelating agent-soluble solids; PGA, polygalacturonic acid. Click here for file
