# Supplementary material for: Analysis of pectin mutants and natural accessions of Arabidopsis highlights the impact of de-methyl-esterified homogalacturonan on tissue saccharification
Source: Biotechnol Biofuels. 2013 Nov 18;6:163. doi: 10.1186/1754-6834-6-163 (PMC3843582; doi:10.1186/1754-6834-6-163)
Supplement: Additional file 1: Figure S1 — Monosaccharide composition of the ChASS fraction of cell walls from WT (Col-0), qua2-1, pme3, PG, and PMEI plants. Monosaccharide composition was determined by HPAEC-PAD. Values are expressed in mol% for each monosaccharide. Bars represent means ± SE (n = 4). Different letters indicate statistically significant differences in each monosaccharides between mutants or transgenic plants and WT plants, according to ANOVA followed by Tukey’s test (P <0.05). ChASS, chelating agent-soluble solids; Col-0, Columbia-0; HPAEC-PAD, high-performance anion-exchange chromatography with pulsed amperometric detection; PG, polygalacturonase; pme3, pectin methylesterase 3; PMEI, pectin methylesterase inhibitor; qua2-1, quasimodo2-1; SE, standard error; WT, wild type. [file 1754-6834-6-163-S1.ppt]

## Slide 1
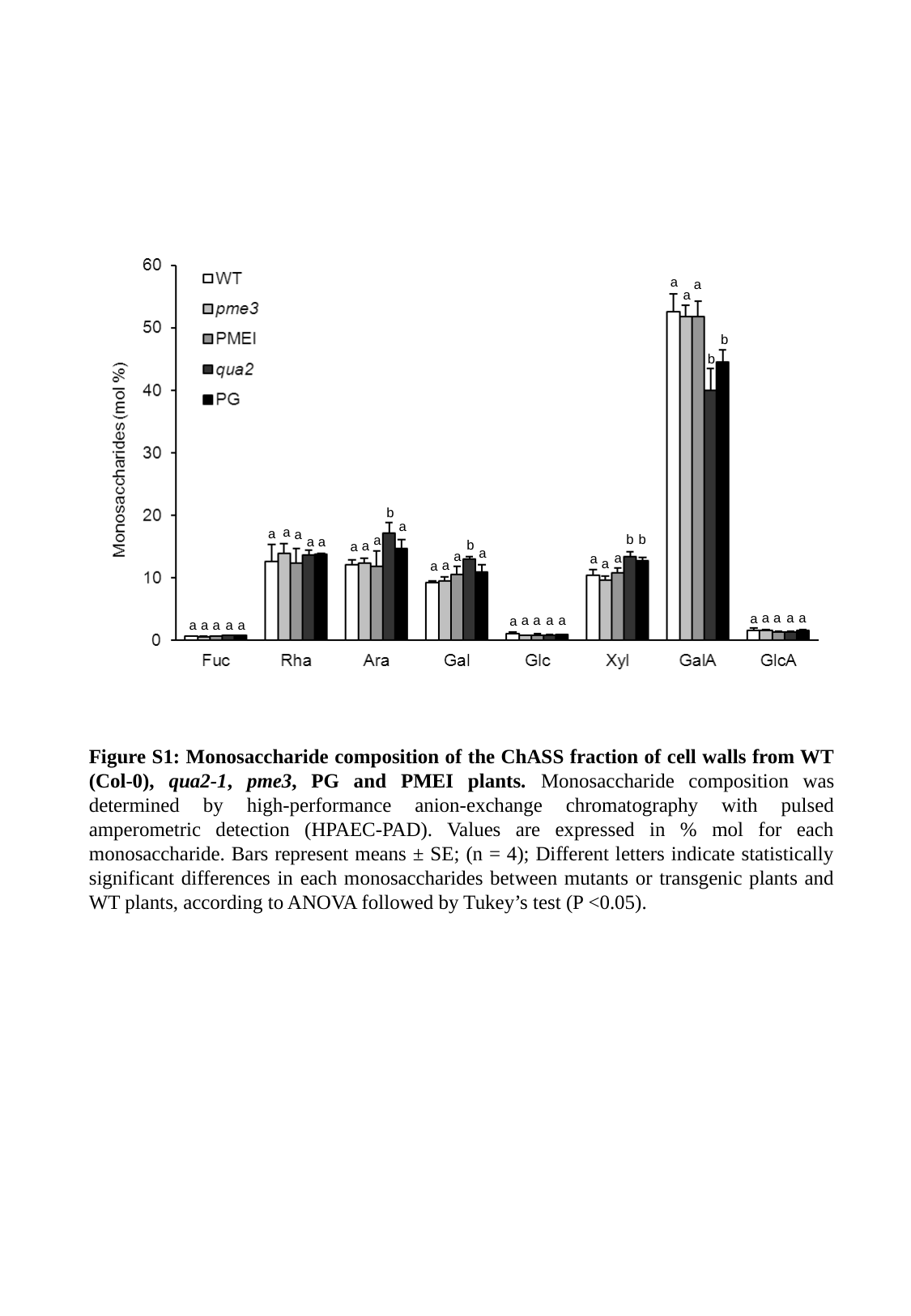

a
a
a
b
b
b
a
a
a
a
b
b
a
a
a
b
a
a
a
a
a
a
a
a
a
a
a
a
a
a
a
a
a
a
a
a
a
a
a
a
Figure S1: Monosaccharide composition of the ChASS fraction of cell walls from WT (Col-0), qua2-1, pme3, PG and PMEI plants. Monosaccharide composition was determined by high-performance anion-exchange chromatography with pulsed amperometric detection (HPAEC-PAD). Values are expressed in % mol for each monosaccharide. Bars represent means ± SE; (n = 4); Different letters indicate statistically significant differences in each monosaccharides between mutants or transgenic plants and WT plants, according to ANOVA followed by Tukey’s test (P <0.05).
