# Supplementary material for: Analysis of pectin mutants and natural accessions of Arabidopsis highlights the impact of de-methyl-esterified homogalacturonan on tissue saccharification
Source: Biotechnol Biofuels. 2013 Nov 18;6:163. doi: 10.1186/1754-6834-6-163 (PMC3843582; doi:10.1186/1754-6834-6-163)
Supplement: Additional file 2: Table S1 — Effect of qua2-1 and pme3 mutations on plant biomass. Fresh weight (FW) of aerial vegetative portion of plants and dry weight (DW/FW) ratio were calculated. Data represent the average ± SD of at least six plants. Asterisks indicate statistically significant differences between mutants and the WT according to Student’s t-test (P <0.05). DW, dry weight; FW, fresh weight; pme3, pectin methylesterase 3; qua2-1, quasimodo2-1; WT, wild type. [file 1754-6834-6-163-S2.ppt]

## Slide 1
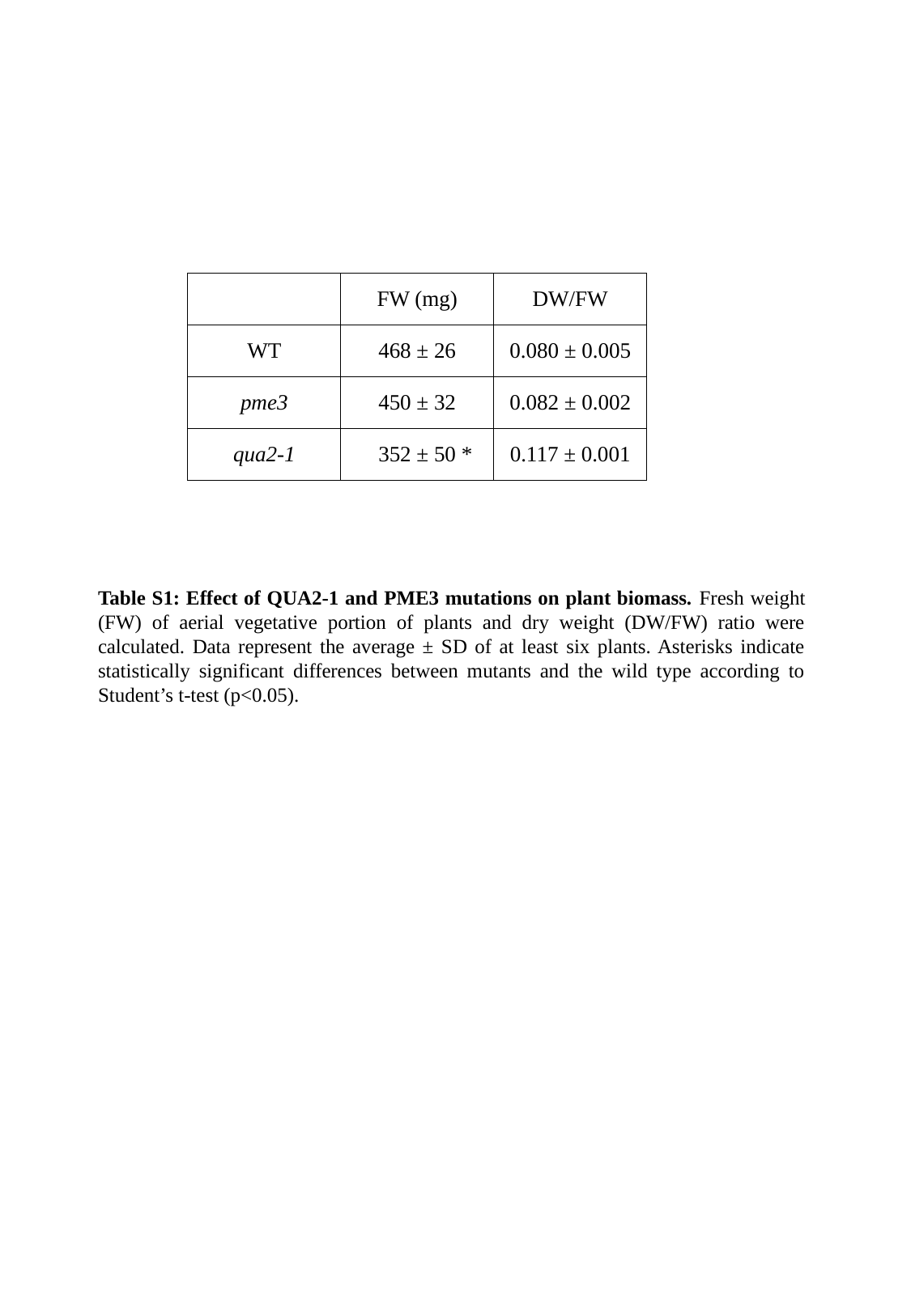

| | FW (mg) | DW/FW |
| --- | --- | --- |
| WT | 468 ± 26 | 0.080 ± 0.005 |
| pme3 | 450 ± 32 | 0.082 ± 0.002 |
| qua2-1 | 352 ± 50 \* | 0.117 ± 0.001 |
Table S1: Effect of QUA2-1 and PME3 mutations on plant biomass. Fresh weight (FW) of aerial vegetative portion of plants and dry weight (DW/FW) ratio were calculated. Data represent the average ± SD of at least six plants. Asterisks indicate statistically significant differences between mutants and the wild type according to Student’s t-test (p<0.05).
