# Supplementary material for: Analysis of pectin mutants and natural accessions of Arabidopsis highlights the impact of de-methyl-esterified homogalacturonan on tissue saccharification
Source: Biotechnol Biofuels. 2013 Nov 18;6:163. doi: 10.1186/1754-6834-6-163 (PMC3843582; doi:10.1186/1754-6834-6-163)
Supplement: Additional file 3: Table S2 — Information about ecotypes included in the nested core collection from INRA including Col-0 and biomass yield. The Versailles identification number (AV), the name of accession, its geographical location, fresh weight (FW) of aerial vegetative portion of plants, and dry weight (DW)/FW ratio are reported. Data represent the average ± SD of at least six plants. Average, standard deviation, and coefficient of variation (CV) calculated from the entire collection are reported. Col-0, Columbia-0; CV, coefficient of variation; DW, dry weight; FW, fresh weight; INRA, National Institute for Agricultural Research. [file 1754-6834-6-163-S3.ppt]

## Slide 1
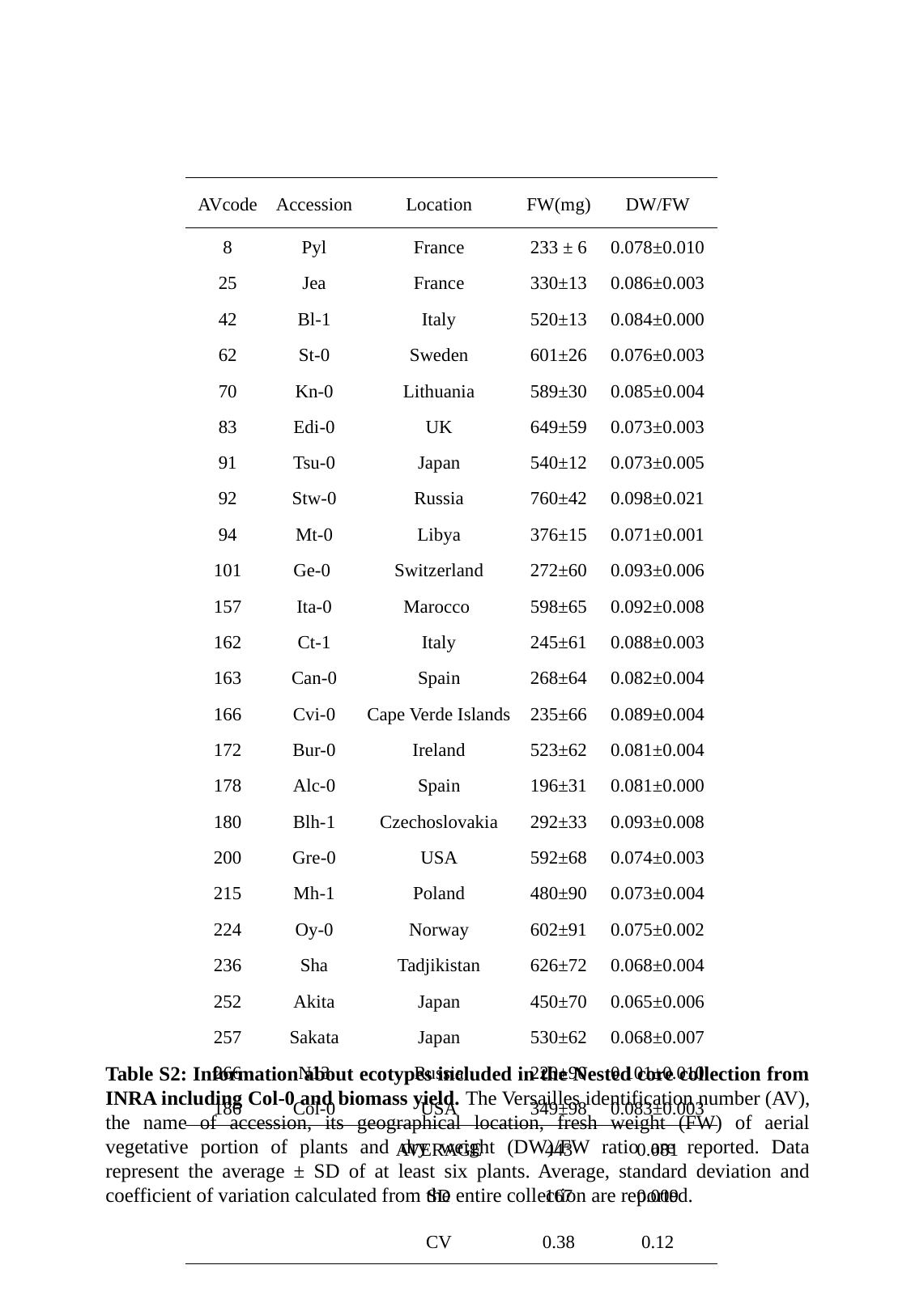

| AVcode | Accession | Location | FW(mg) | DW/FW |
| --- | --- | --- | --- | --- |
| 8 | Pyl | France | 233 ± 6 | 0.078±0.010 |
| 25 | Jea | France | 330±13 | 0.086±0.003 |
| 42 | Bl-1 | Italy | 520±13 | 0.084±0.000 |
| 62 | St-0 | Sweden | 601±26 | 0.076±0.003 |
| 70 | Kn-0 | Lithuania | 589±30 | 0.085±0.004 |
| 83 | Edi-0 | UK | 649±59 | 0.073±0.003 |
| 91 | Tsu-0 | Japan | 540±12 | 0.073±0.005 |
| 92 | Stw-0 | Russia | 760±42 | 0.098±0.021 |
| 94 | Mt-0 | Libya | 376±15 | 0.071±0.001 |
| 101 | Ge-0 | Switzerland | 272±60 | 0.093±0.006 |
| 157 | Ita-0 | Marocco | 598±65 | 0.092±0.008 |
| 162 | Ct-1 | Italy | 245±61 | 0.088±0.003 |
| 163 | Can-0 | Spain | 268±64 | 0.082±0.004 |
| 166 | Cvi-0 | Cape Verde Islands | 235±66 | 0.089±0.004 |
| 172 | Bur-0 | Ireland | 523±62 | 0.081±0.004 |
| 178 | Alc-0 | Spain | 196±31 | 0.081±0.000 |
| 180 | Blh-1 | Czechoslovakia | 292±33 | 0.093±0.008 |
| 200 | Gre-0 | USA | 592±68 | 0.074±0.003 |
| 215 | Mh-1 | Poland | 480±90 | 0.073±0.004 |
| 224 | Oy-0 | Norway | 602±91 | 0.075±0.002 |
| 236 | Sha | Tadjikistan | 626±72 | 0.068±0.004 |
| 252 | Akita | Japan | 450±70 | 0.065±0.006 |
| 257 | Sakata | Japan | 530±62 | 0.068±0.007 |
| 266 | N13 | Russia | 220±90 | 0.101±0.010 |
| 186 | Col-0 | USA | 349±98 | 0.083±0.003 |
| | | AVERAGE | 443 | 0.081 |
| | | SD | 167 | 0.009 |
| | | CV | 0.38 | 0.12 |
Table S2: Information about ecotypes included in the Nested core collection from INRA including Col-0 and biomass yield. The Versailles identification number (AV), the name of accession, its geographical location, fresh weight (FW) of aerial vegetative portion of plants and dry weight (DW)/FW ratio are reported. Data represent the average ± SD of at least six plants. Average, standard deviation and coefficient of variation calculated from the entire collection are reported.
