# Supplementary material for: Analysis of pectin mutants and natural accessions of Arabidopsis highlights the impact of de-methyl-esterified homogalacturonan on tissue saccharification
Source: Biotechnol Biofuels. 2013 Nov 18;6:163. doi: 10.1186/1754-6834-6-163 (PMC3843582; doi:10.1186/1754-6834-6-163)
Supplement: Additional file 4: Figure S2 — Quantification of uronic acids and degree of methylesterification (DM) in cell walls of the Arabidopsis core collection. (A) Uronic acid content and (B) degree of methylesterification (DM) were analyzed in cell walls extracted from 4-week-old fully expanded leaves. Bars represent averages ± SD (n = 10). Different letters indicate statistically significant differences, according to ANOVA followed by Tukey’s test (P <0.05). DM, degree of methylesterification. [file 1754-6834-6-163-S4.ppt]

## Slide 1
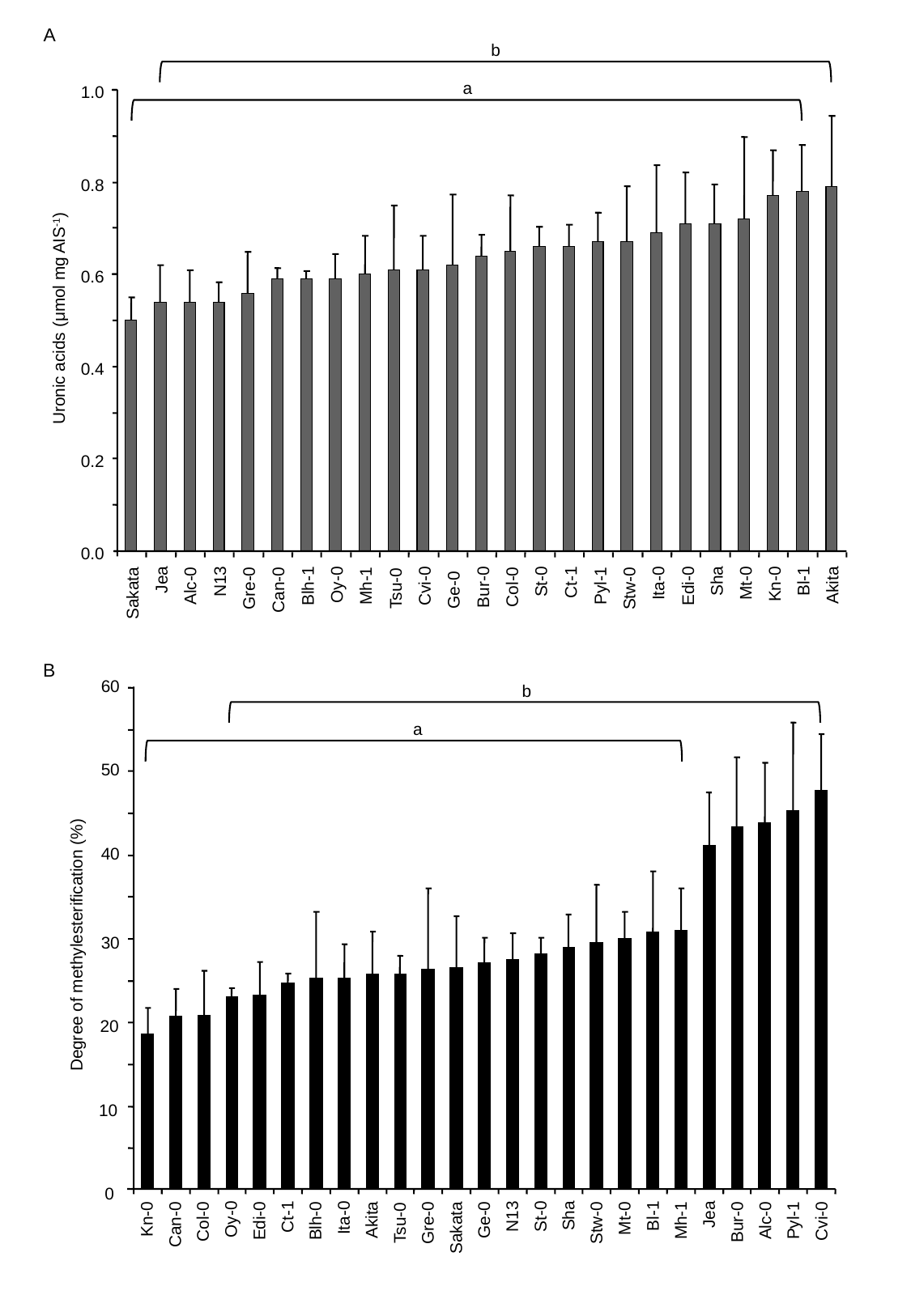

A
b
a
1.0
0.8
0.6
Uronic acids (μmol mg AIS-1)
0.4
0.2
0.0
Jea
Sha
Bl-1
N13
St-0
Ct-1
Ita-0
Mt-0
Kn-0
Oy-0
Akita
Alc-0
Mh-1
Pyl-1
Blh-1
Edi-0
Cvi-0
Col-0
Bur-0
Ge-0
Gre-0
Tsu-0
Stw-0
Can-0
Sakata
B
b
60
a
50
40
30
Degree of methylesterification (%)
20
10
0
Jea
Sha
Bl-1
N13
St-0
Ct-1
Ita-0
Mt-0
Kn-0
Oy-0
Akita
Ge-0
Mh-1
Alc-0
Pyl-1
Edi-0
Blh-0
Cvi-0
Col-0
Bur-0
Stw-0
Tsu-0
Gre-0
Can-0
Sakata

## Slide 2
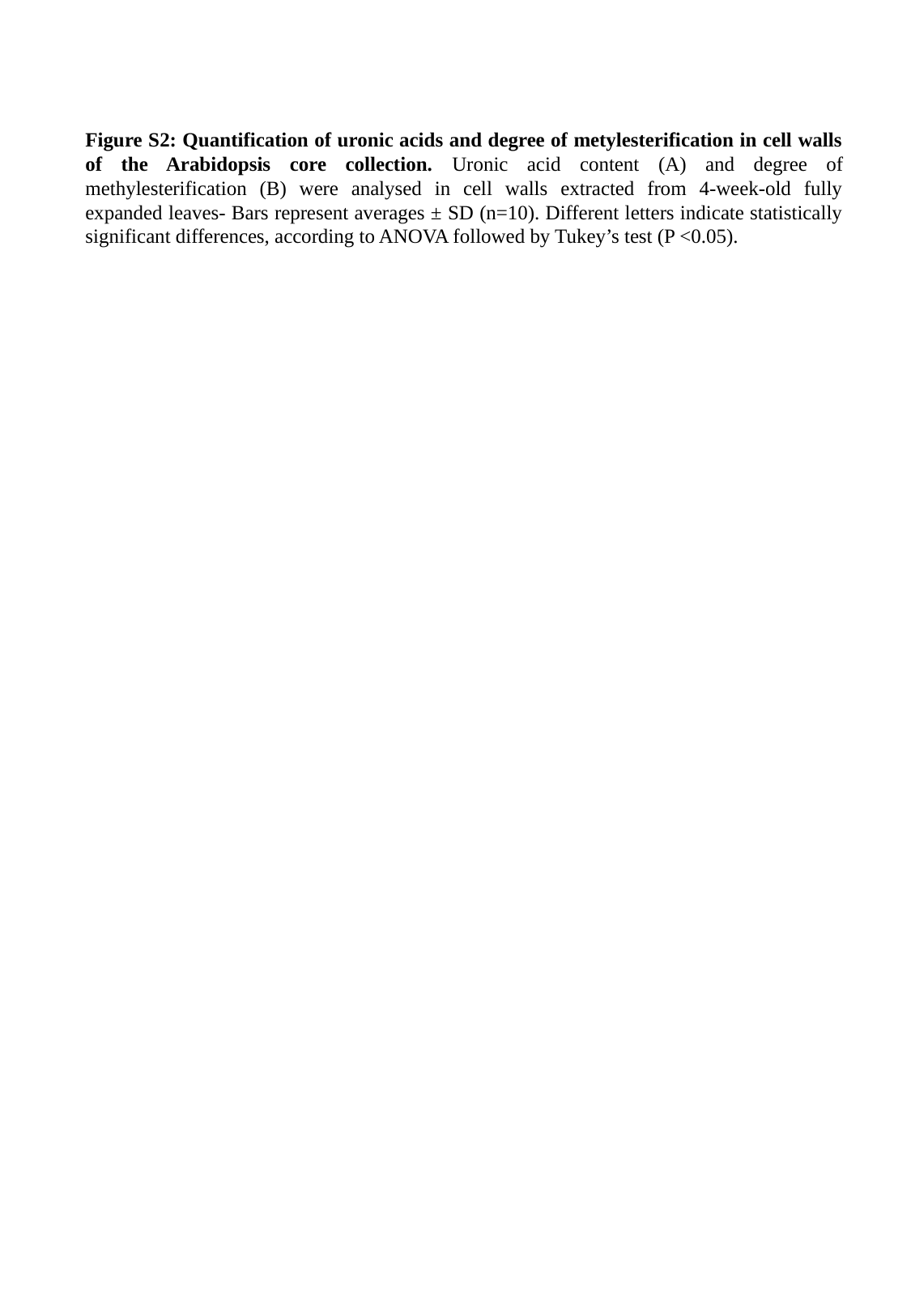

Figure S2: Quantification of uronic acids and degree of metylesterification in cell walls of the Arabidopsis core collection. Uronic acid content (A) and degree of methylesterification (B) were analysed in cell walls extracted from 4-week-old fully expanded leaves- Bars represent averages ± SD (n=10). Different letters indicate statistically significant differences, according to ANOVA followed by Tukey’s test (P <0.05).
