# Supplementary material for: Analysis of pectin mutants and natural accessions of Arabidopsis highlights the impact of de-methyl-esterified homogalacturonan on tissue saccharification
Source: Biotechnol Biofuels. 2013 Nov 18;6:163. doi: 10.1186/1754-6834-6-163 (PMC3843582; doi:10.1186/1754-6834-6-163)
Supplement: Additional file 5: Figure S3 — Monosaccharide composition of the ChASS fraction of cell walls from Col-0, Edi-0, and Pyl-1. Monosaccharide composition was determined by HPAEC-PAD. Values are expressed in mol% for each monosaccharide. Bars represent means ± SE (n = 4). Different letters indicate statistically significant differences in each monosaccharides of Edi-0 and Pyl-1, with respect to Col-0 according to ANOVA followed by Tukey’s test (P <0.05). ChASS, chelating agent-soluble solids; Col-0, Columbia-0; HPAEC-PAD, high-performance anion-exchange chromatography with pulsed amperometric detection; SE, standard error. [file 1754-6834-6-163-S5.ppt]

## Slide 1
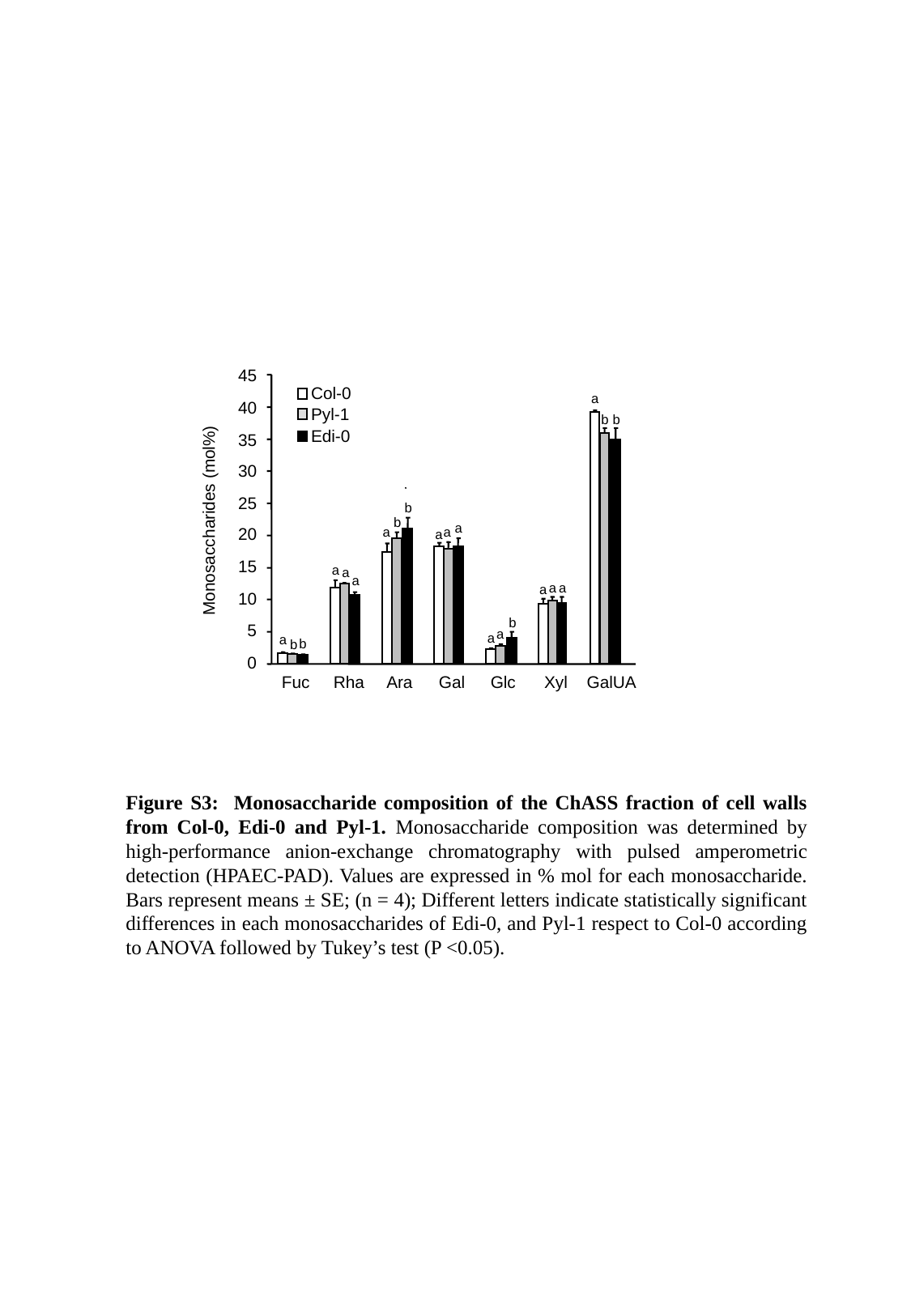

45
Col-0
a
40
Pyl-1
b
b
Edi-0
35
30
25
b
b
Monosaccharides (mol%)
a
a
a
a
20
a
15
a
a
a
a
a
10
b
a
5
a
a
b
b
0
Fuc
Rha
Ara
Gal
Glc
Xyl
GalUA
Figure S3: Monosaccharide composition of the ChASS fraction of cell walls from Col-0, Edi-0 and Pyl-1. Monosaccharide composition was determined by high-performance anion-exchange chromatography with pulsed amperometric detection (HPAEC-PAD). Values are expressed in % mol for each monosaccharide. Bars represent means ± SE; (n = 4); Different letters indicate statistically significant differences in each monosaccharides of Edi-0, and Pyl-1 respect to Col-0 according to ANOVA followed by Tukey’s test (P <0.05).
