# Supplementary material for: Analysis of pectin mutants and natural accessions of Arabidopsis highlights the impact of de-methyl-esterified homogalacturonan on tissue saccharification
Source: Biotechnol Biofuels. 2013 Nov 18;6:163. doi: 10.1186/1754-6834-6-163 (PMC3843582; doi:10.1186/1754-6834-6-163)
Supplement: Additional file 6: Figure S4 — ChASS arrays using PAM1 antibodies. (A) Representative arrays relative to different Arabidopsis accessions are shown. The numbers represent three independent replicates for each accession. ChASS fractions were applied as 1 μL aliquots in a threefold dilution series (10, 3, and 1 μg/μL). As standard, PGA was also applied as 1 μL aliquots in a dilution series (3, 1, and 0.3 μg/μL). (B) Schematic representation of quantification steps of PAM1 signal, using the immunoblot for the N13 accession (A). ImageJ software was used to quantify spot signals. The optical density of each spot was quantified as the peak area and defined as PAM1 signal. ChASS, chelating agent-soluble solids; PGA, polygalacturonic acid. [file 1754-6834-6-163-S6.pdf]

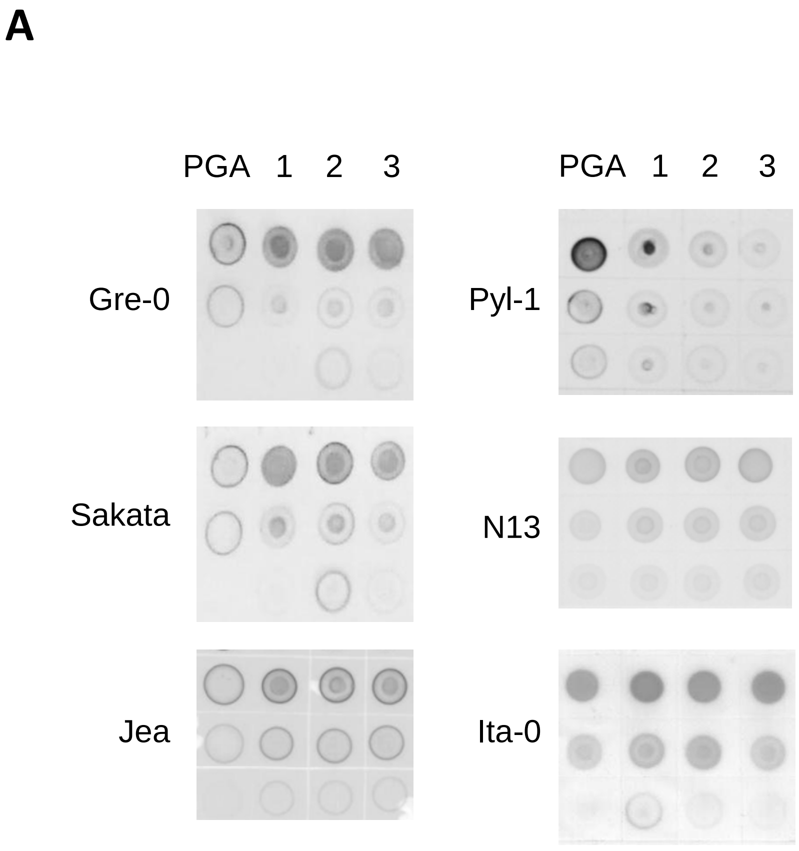

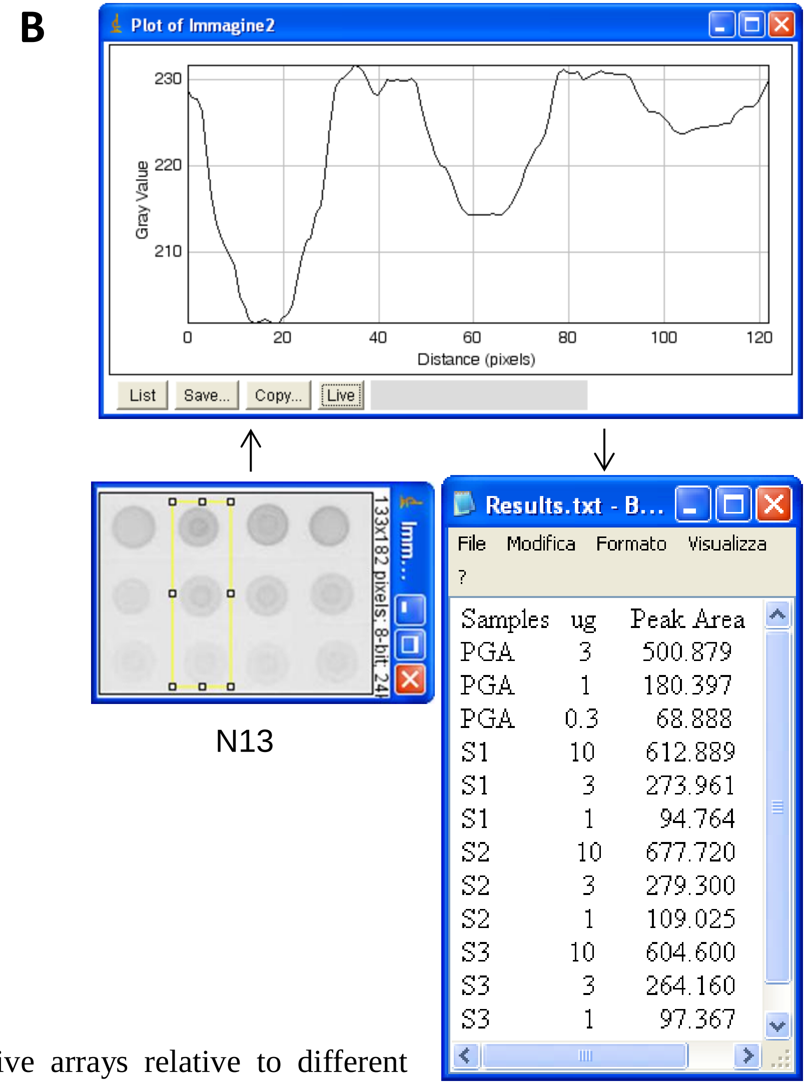

Figure S4 ChASS arrays using PAM1 antibodies. A) Representative arrays relative to different Arabidopsis accessions are shown. The numbers represent three independent replicates for each accession. ChASS fractions were applied as 1 µL aliquots in a 3-fold dilution series (10, 3, and 1 µg/µL). As standard, PGA was also applied as 1 µL aliquots in a dilution series (3, 1 and 0.3 µg/µL). B) Schematic representation of quantification steps of PAM1 signal, using the immuoblot for the N13 accession (A). ImageJ software was used to quantify spot signals. The optical density of each spot was quantified as peak area and defined as PAM1 signal.
